# Oncogenic human papillomavirus genital infection in southern Iranian women: population-based study versus clinic-based data

**DOI:** 10.1186/1743-422X-9-194

**Published:** 2012-09-12

**Authors:** Seyed Sajjad Eghbali, Roya Amirinejad, Narges Obeidi, Shiva Mosadeghzadeh, Katayoun Vahdat, Fatemeh Azizi, Raha Pazoki, Zahra Sanjdideh, Zahra Amiri, Iraj Nabipour, Keivan Zandi

**Affiliations:** 1 Department of Virology, The Persian Gulf Tropical Medicine Research Center, Bushehr University of Medical Sciences, Bushehr, Iran; 2 Department of Pathology, School of Medicine, Bushehr University of Medical Sciences, Bushehr, Iran; 3 Department of Biochemistry, The Persian Gulf Marine Biotechnology Research Center, Bushehr University of Medical Sciences, Bushehr, Iran; 4 Department of Medical Microbiology, Faculty of Medicine, Tropical and Infectious Disease Research and Education Center, University of Malaya, Kuala Lumpur, Malaysia

**Keywords:** Human papilloma virus, Bushehr, Cervical cancer, Iran, PCR

## Abstract

**Background:**

Epidemiological studies on genital human papilloma viruses infection (HPVs) in general population are crucial for the implementation of health policy guidelines for developing the strategies to prevent the primary and secondary cervical cancer. In different parts of Iran, there is a lack of population-based studies to determine the prevalence of HPV in the general population. The aim of this population-based study is to compare the prevalence rate of genital HPV infection among reproductive women with our previous clinic-based data, which showed a prevalence rate of 5% in women in southern Iran.

**Results:**

Using general primers for all genotypes of HPV, of 799 randomly selected women, five (0.63%, 95% CI 0.23-1.55%) tested positive for HPV DNA. Overall, seven different HPV genotypes were detected: six types (16, 18, 31, 33, 51 and 56) were carcinogenic, or “high risk genotypes” and one genotype (HPV-66) was “probably carcinogenic.”

**Conclusions:**

In a population-based study, the prevalence of HPV infection among southern Iranian women was lower than that observed worldwide. However, our gynaecological clinic-based study on the prevalence of HPV infection showed results comparable with other studies in the Middle East and Persian Gulf countries. Since gynaecological clinic-based data may generally overestimate HPV prevalence, estimates of prevalence according to clinic-based data should be adjusted downward by the population-based survey estimates.

## Background

Cervical cancer is an important public health problem worldwide, especially in developing countries, where more than 80 percent of the estimated global cases occur [1,2]. Persistent infection by certain types of human papillomavirus (HPV) is a principal risk for the development of both high-grade cervical cancer precursors and invasive cervical cancer [3,4].

Oncogenic HPV DNA is detected in more than 90 percent of the cervical tissues of patients with cervical carcinoma or cervical intraepithelial neoplasia [5,6]. Worldwide, 70% of cervical cancers are due to oncogenic HPV 16 and 18 genotypes [7]. In Iran, HPV infection is prevalent in all categories of cervical neoplasia [8]. The prevalence of HPV in cervical cancer in Iran was reported to be similar to that in most countries of the world [9].In southern Iranian patients with cervical cancer, more than 80% of samples were HPV DNA positive [10]. The HPV type 16 was also the most common genotype found in Iranian women with cervical cancer [9].

Most epidemiological data for HPV infection comes from cross-sectional studies with samples taken from women in different hospitals and gynecological clinics [8-14]. These women had cervical neoplasia or cancer [8-10], abnormal cervical cytology [11-13], and normal Pap smears [10,11,14,15].

Regarding the widespread use of HPV vaccines against oncogenic HPV types that potentially reduce the incidence of cervical and other anogenital cancers, Data on the HPV burden in the general population is essential to strengthen the guidelines for the implementation of health policy strategies for the prevention of primary and secondary cervical cancer [16].

Therefore, we carried out The purpose of this population-based study is to investigate the HPV burden among women in southern Iran Since we are particularly interested in understanding the level of and the difference in the prevalence of HPV infection between our population-based study and our clinic-based data, we compared the results of our previous clinic-based study with those of the present study.

## Methods

### Community sampling, survey procedure and data collection

This study was approved by the medical-ethical committee of Bushehr University of Medical Sciences.

A cross-sectional, population-based study was used to select women of childbearing age (21–50 years old) from Bushehr Port (the center of Bushehr province, an Iranian province with the longest border on the Persian Gulf) from November 2009 to May 2010.

Participants were an age-stratified random sample of women aged 21 to 50 years. They were selected from 51 urban and 24 suburban areas in Bushehr Port. The first cluster in each area was selected using a random number table. The sampling interval was added sequentially to the random number to complete the selection of the remaining clusters in each area. The total number of women selected in each area was proportional to the total number of childbearing women in that area.

Regarding our previous study on women who had routine Pap tests in Bushehr Port [12], it was estimated that the prevalence of HPV infection in a population-based setting was unlikely to exceed 5% (p = 0.05). With a confidence level of 95% and absolute precision of 2 percentage points (d = 0.02) and using a simple random sampling method, the sample size was estimated to be around 456 women. By considering a design effect of 2 due to the cluster sampling strategy of the study, the estimated sample size was doubled (n = 912 women). Participants who were pregnant or menstruating at the time of the tests were excluded. In Iranian-Islamic culture, sexual intercourse before marriage is forbidden. Therefore, women who had never been married were considered virgins and were also excluded.

The selected participants were informed about the study through a letter distributed door-to-door by the survey groups employed in the study. After briefing the respondents to the letter with some basic information about cervical cancer and its associated risk factors, they were invited to participate in the screening program at the Persian Gulf Tropical Medicine Research Center at Bushehr University of Medical Sciences.

All participants were asked to come to the survey centre at 07:30 am and stay until 9:30 am. Upon their arrival, information regarding age, marital status, education, socioeconomic status, smoking, obstetrics history, history of oral contraceptive usage, and sexually transmitted diseases were recorded on a questionnaire used by trained interviewers.

Written informed consent was obtained from the all participants for publication of this report.

Based on national reports, 200,000 Tomans (200 US $) income per month was defined as the line for poverty. The women below the poverty line or in the range which households could maintain a minimum standard of living (200,000 to 450,000 Tomans income per month) were considered to be in the low socioeconomic status.

Two separate samples were taken from the ectocervix and endocervix using a wooden Ayre spatula (for cytopathological exam) and endocervical cytobrush (for the PCR test).

The Pap smear slides were immediately fixed with ethanol and further processed for diagnosing by cytopathologists. Bethesda System 2001 for reporting cervical/endocervical/vaginal cytology (Pap smears) was used (http://www.bethesda2001.cancer.gov). Cells showing koilocytic change, multinucleation, and dyskeratosis, with the addition of anucleate keratinised squamous cells and keratinised squamous were considered cytological changes of HPV infection.

### DNA extraction

The exfoliated cells on the cytobrush were suspended in Tris-EDTA, and the suspension was centrifuged at 3000 g for 5 minutes. The supernant was removed, and the cell pellet was re-suspended in 0.5 ml of Tris-EDTA and then transferred to the sterile microotube. All samples were digested by lysis buffer containing proteinase K, which was followed by extensive extraction with phenol/chloroform [17]. The DNA quality was evaluated by a PCR test using following primers: forward primer PCO3: 5′-ACACAACTGTGTTCACTAGC-3′; reverse primer PCO4: 5′-CAACTTCATCCACGTTCACC-3′. These primers can amplify a 110 bp product from the human β-Globin gene [18].

### HPV PCR

β-Globin positive samples were subjected to HPV PCR by forward primer GP5+: 5^′^-TTTGTTACTGTGGTAGATACTAC-3^′^ and reverse primer GP6+:5′-AAAAATAAACTGTAAATCATATTC-3^′^ for L1 open reading frame (ORF), which amplifies a 150 bp product from the HPV L1 ORF. The validity of the PCR test was approved in previous studies [12,17,18]. PCR was performed according to the procedure described previously [18]. The DNA extracted from the HeLa cell line was used as the HPV positive control and no DNA was added for the negative control. Samples were subsequently subjected to agarose gel electrophoresis.

### HPV genotyping by reverse hybridization using the INNO-LiPA HPV

#### Genotyping system

The DNA extracted from the HPV PCR positive samples were subjected to genotyping using the INNO-LiPA HPV genotyping system (INNOGENETICS N.V., Ghent, Belgium) at the Keyvan Virology Laboratory, Tehran, Iran. Briefly, the 28 oligonucleotide probes that recognize 25 different types (Table 1) were tailed with poly(dT) and immobilized as parallel lines to membrane strips. The interpretation for the HPV genotyping assay was performed as described previously [19].

**Table 1:** The prevalence of HPV DNA positivity and cervical cytopathological findings of Iranian women according to the type of study

	**Population-based study**	Clinic-based study*
	**n = 799**	**n = 200**
PCR Positive for HPV DNA; n (%)	5 (0.63%)	11 (5.5%)
Pap test positive for HPV; n(%)	9 (1.12%)	9 (4.5%)
PCR and Pap Test Positive for HPV; n	2	4
Carcinogenic HPV genotypes	16, 18, 31, 33, 51, 56	16, 18
Probably carcinogenic HPV genotypes	66	53

*Zandi et al. [12].

### Statistical analysis

All statistical analyses were performed using the PASW Statistics GradPack 18 (SPSS Inc., Chicago, IL).

## Results

### Characteristics of the studied population

A total of 799 women of reproductive age, 17 to 50 years, were evaluated. The mean (± SD) age of women in the study was 35.96 (± 8.08). Of 799 participants, 26.3% were between 17 and 30 years, 36.5% were between 31 and 40 years, and 37.2% were between 41 and 50 years.

The educational level of 112 (15.07%) women was elementary school, and 33 (4.44%) women were illiterate. A total of 112 (15.07%) participants had low socioeconomic status.

The majority of the studied population (98.7%) was married. The mean age of first sexual intercourse (during marriage) was 19.16 ± 3.97 years. None of the women had a history of multiple sexual partners. The median number of pregnancies was three for the studied population. The history of tobacco smoking was found in 14.4% of the women. A total of 464 (58.1%) women had used oral contraceptive pills. However, 124 (15.5%) of the studied population reported that they were current users of oral contraceptives. The median duration of oral contraceptive usage by the participants was three years.

Previous sexually transmitted disease was reported by 44 (5.5%) women. However, 11 (1.4%) women reported a positive history of previous sexually transmitted disease via their husbands. Of the studied population, 375 (46.9%) had never had a Pap test.

### HPV DNA PCR and genotyping

Using general primers for all genotypes of HPV, of 799 tested samples by PCR, five (0.63%, 95% CI 0.23-1.55%) women were positive for HPV DNA.

Genotyping of the PCR positive samples revealed that two women were infected with more than one genotype (one of them infected with genotypes 16, 56 and 66, and the other one infected with genotypes 31 and 33). The other three patients were infected with one genotype of HPV (16, 51 or 18). Therefore, seven different HPV genotypes were detected, among which six types (16, 18, 31, 33, 51 and 56) were carcinogenic, or “high risk genotypes” and one genotype (HPV-66) was “probably carcinogenic.” Table 1 shows the detected HPV genotypes. The median age of the HPV-positive women was 25 years (min = 24, max = 42 years). All had been married when they were less than 24 years old. None had a history of multiple sexual partners, previous sexually transmitted disease, or tobacco smoking. All had been pregnant, and the median of parity was (min = 0, max = 3). Four out of five HPV-positive women had a history of oral contraceptive use. Only one woman had ever had a Pap test. Two HPV-positive women had low socioeconomic status. However, none of the HPV-positive women was illiterate.

### Cytopathology

The Pap tests of 40 (5.0%) women showed epithelial cell abnormalities. Atypical squamous cells of undetermined significance (ASCUS) and cervical intraepithelial neoplasia (CINI) were found in 32 (4.0%) and eight (1.0%) participants, respectively. Cellular changes associated with HPV were detected in nine (1.12%) women. However, two were found to be HPV-positive by PCR. On the other hand, three women who were identified as HPV DNA positive by the PCR method were negative for HPV infection when they were evaluated by the Pap test.

## Discussion

The overall prevalence of HPV infection among the women in our study is lower than that observed worldwide. To the best of our knowledge, this is the first population-based study in the south part of Iran to investigate the prevalence rate of HPV infection in this region.

The prevalence rate of HPV infection differs notably according to geographical region [1]. The worldwide prevalence of HPV infection in women with normal cervical cytology was estimated to be 11.7% [20]. Based on molecular epidemiologic evidence among asymptomatic women in the general population, the prevalence of HPV infection was estimated to be in the range of 2-44% [21]. In a comprehensive review, Seoud et al. reported that HPV was found in 1.5-13% of the general population of the Middle East and the Persian Gulf countries [22]. Our study shows that the prevalence of HPV infection in southern Iranian women is among the lowest in the world and Middle Eastern countries. This prevalence rate is much lower than that reported for the Persian Gulf region (4-11%) [22].

Comparisons of HPV prevalence by geographical area or country are generally hampered by the heterogeneity in laboratory assays employed for HPV infection detection, the population included (women with normal cytology, population-based surveys, or women in routine screening programs), and the variation in HPV types detected [1].

Although a few population-based studies on the prevalence of HPV infection in the Persian Gulf and the Middle Eastern countries could be found in the medical literature, the low prevalence of oncogenic HPV infection in Iran (the current study) and Kuwait (1.39%) [23] compared with the rest of the world, may reflect differences in sexual behavior across the Persian Gulf region. In both countries, the majority of women have their first experience of sexual intercourse during married life and have one husband in a lifetime [23]. However, questions related to sexual and reproductive behavior are considered taboo in Muslim countries.

The present community study did not confirm our previous study on reporting HPV prevalence among women in southern Iran [12] despite of using the same protocols and techniques. However, there is a difference between their target population (population-based study versus gynecology clinic-based study).

In the previous study, we determined the prevalence of various HPV genotypes among women who had routine Pap smear tests in a university gynecology clinic (Table 1). In that study, the prevalence of various HPV genotypes was 11 (5.5%) in 200 samples [12]. This prevalence rate was similar to those reported from various studies in Iran. All previous studies in Iran regarding the prevalence of HPV infection were performed on women attending gynaecological clinics for routine screening programs and examinations [11-13]. HPV DNA was identified in 5.7% women who were admitted to different hospitals and gynecological clinics in Tehran [13]. Safaei et al. [14] reported positive HPV findings in 5.5% of women who were examined at two gynecological clinics in Shiraz, a city in the southwest of Iran with a population very similar to the population in the current study. Therefore, it seems that the prevalence of HPV infection was very similar in different gynecological clinics in Iran and was comparable to other geographical areas in Muslim and Middle Eastern countries.

Although, in a recent population based study on genital HPV by Khodakarami and her colleagues [15] among 2342 women in one of the densely populated suburb of Theran the prevalence of HPV infection was 7.8% which is different from our present finding. The number of participant as well as the differences between their communities might be the reason of obsereved difference. Indeed, they have used different techniques compared to ours with different sensitivity such as DNA extraction using magnetic beads and liquid based cytology and even different type of sampling and it could be also considered as the other factor that would explain the difference between our data and their findings.

Similarly, the observed prevalence of HPV high and low risk types in cervical samples taken in a population-based study in Italy was lower than those in studies that were performed on women undergoing voluntary cervical cancer screening in gynaecological clinics [24,25]. Notably, sampling in gynaecological clinics or voluntary screening programs may select women who are more sexually active than the general population or those with abnormal cytological results. These women usually seek more cervical examinations than women with normal cytology [25]. In contrast, the design of a population-based study mandates a better coverage of the general population [26].

Based on the present study nine samples were detected HPV positive by Pap smear test meanwhile two of them were approved by HPV DNA PCR test. Moreover, there are other three HPV DNA positive samples among the Pap smear test negatives. This observation could be because of the lack of specificity of Pap smear test for HPV detection.

Previous studies have indicated a significant geographic variation in the pattern of oncogenic HPV types. HPV 16 was the most commonly identified type in most countries of the world [27]. In the current study and our previous clinic-based study, HPV 16 was the most prevalent type, and HPV 18 was the second most prevalent oncogenic HPV type. These findings are consistent with studies on the Extended Middle East and North Africa (EMENA) [22]. It has been estimated that 32% of about 291 million women worldwide who are carriers of HPV are infected with HPV 16 or HPV 18, or both [20]. HPV types 58 and 52 play a more prominent role in cervical cancer in all regions of Asia—except India and Iran—than in other parts of the world [8,28-30]. We also did not find these oncogenic HPV types in our both studies.

A comparison of our results with some similar studies in Muslim and Middle Eastern countries [31,32] showed a similar prevalence of epithelial abnormalities in Pap smears. In the current study, the Pap tests of 40 (5.0%) women showed epithelial cell abnormalities. Overall, the majority of women had negative results of both HPV DNA and Pap tests. Also, weakness of cytopathological detection of genital HPV was noted by Khodakarami et al. [15]. Therfore, re-screening of those women can be done at three years [33]. The extended period of re-screening for women with negative results for both tests is considered an advantage for HPV DNA testing [3]. Indeed, the type of used device for cytopathology specimen collection (wooden Ayre spatula) might be a reason for poor quality of detection compared to the plastic cytobrush that used for PCR test.

Our study has several limitations. The small sample size of this study in a population-based setting may have underestimated the prevalence of HPV infection in the general population. Indeed, this limitation may decrease the chance of detection of different genotypes with lower prevalence such as HPV-42 and HPV-6 compared to the prevalent genotypes. In addition, the participation rate in the current study was 87.60%. The non-responders may produce a selection bias. It is probable that the non-responders may be at higher risk of HPV acquisition. However, the major reasons for non-participation were having to work, anxiety and doing Pap smear examination before the invitation time. The cross-sectional design of the study did not provide the opportunity to examine trends over time. We also did not evaluate risk factors for the acquisition and persistence of oncogenic HPV types. We also did not have information on the husband’s sexual behavior and therefore potential confounding effects cannot be evaluated in our study. Because of the low prevalence of HPV infection in the current study, we cannot evaluate the prevalence of oncogenic HPV types among women with epithelial abnormalities in cervical cytological examinations. Moreover, The small number of HPV positive cases in our study precludes any age-specific incidence rate.

Thus, larger population-based studies in different regions are needed to eliminate the drawbacks of our study and to determine the accurate prevalence of HPV infection among women in Iran. Based on the very low prevalence of HPV infection in this study, there are currently no indicative data to support use of HPV vaccine in southern Iranian women. Since cervical cancer ranks as 12th most frequent cancer among women in Iran [16], screening with HPV plus Pap tests every 2 or 3 years may save additional years of life at reasonable costs.

## Conclusions

In conclusion, in a population-based study, the prevalence of HPV infection among southern Iranian women was very low. Indeed, the prevalence of HPV was significantly lower than we reported in our clinic-based study. In other words, gynaecological clinic-based data generally overestimate HPV prevalence. Therefore, estimates of prevalence based on clinic-based data should be adjusted downward according to population-based survey estimates [25]. Undoubtedly, estimation of HPV infection based on population-based surveys would provide valuable data particularly to intoduce the strategies for the screening of cervical cancer and HPV vaccination in Iran.

## Competing interests

The authors declare that they have no competing interests.

## Authors’ contributions

RA and NO designed and conceived the PCR test. SSE performed the pap smear test. SM, FA, ZA and RP have collected the samples. ZS co operated as lab staff for PCR performing. IN, KV and KZ conceived the whole study and edited the manuscript. We confirm that all authors read and approved the final manuscript.
